# Waist-based anthropometric measures and central adiposity-related comorbidities in children

**DOI:** 10.4102/safp.v66i1.5932

**Published:** 2024-08-14

**Authors:** Howard Gomwe, Lesego Phiri, Chioneso S. Marange

**Affiliations:** 1 Skills Centre, Faculty of Health Sciences, Sefako Makgatho Health Sciences University, Pretoria, South Africa; 2 Department of Statistics, Faculty of Science, University of Fort Hare, East London, South Africa

**Keywords:** central adiposity, waist circumference, waist-hip ratio, waist-to-height ratio, rural children

## Abstract

**Background:**

Waist-related measures are commonly used to classify central adiposity and related comorbidities. This classification may be essential among children, as it may identify the risk of future non-communicable diseases.

**Methods:**

A cross-sectional study was conducted in the Eastern Cape province, South Africa, among 459 primary school learners aged 9–14 years. Height, weight and waist circumference (WC) were measured using standardised techniques recommended by World Health Organization (WHO). The anthropometric measurements, including body mass index (BMI), WC, waist-to-hip ratio (WHR) and waist-to-height ratio (WHtR) were computed and evaluated.

**Results:**

Most participants were girls (57.70%) with an average age of 11.20 ± 1.60 years. The average weight was 38.81 kg ± 10.49 kg with an average height of 144.16 (standard deviation [s.d.] = 10.37) cm. The sample had a BMI of 18.41 kg/m^2^ (s.d. = 3.19). The results showed, on average, WC of 62.10 cm ± 8.12 cm, WHR of 0.82 ± 0.15 and WHtR of 0.44 ± 0.05. Girls reported significantly higher BMI, WC and WHtR. Based on WHtR, the results showed the acceptable ability to classify children according to abdominal obesity, thus identifying their risk for comorbidities.

**Conclusion:**

Overall body fat indicated by BMI and central obesity shown by waist-related anthropometric measures can play a significant role in classifying children in terms of their risk of comorbidities.

**Contribution:**

To prevent the risks of metabolic diseases in childhood, it is necessary to detect abdominal obesity early using WC-based anthropometric measurements, especially WHtR, to identify those at risk.

## Introduction

Childhood obesity is becoming a global public health problem in both developing and developed countries and has shown a significant increase in recent decades.^1,2^ It is a major contributory factor for several severe chronic diseases, such as cardiovascular disease (CVD) and type 2 diabetes mellitus (T2DM).^1,3^ Being overweight or obese has become more common in South Africa, which is concerning because having a high body mass index (BMI) increases the risk of developing numerous non-communicable diseases (NCDs).^4^ Several children are being affected by these chronic diseases when they are still young, with high risks of suffering from them throughout their lives.^5^ To prevent the problem of childhood obesity and overweight, it is necessary to have early detection of metabolic diseases.

Body mass index is a commonly used measure of body fat in children and adults. However, it does not distinguish between lean and fat mass and is a poor predictor of cardiometabolic risks.^6^ It is evident that central adiposity bears more health risks than total obesity measured by BMI. Therefore, it is ideal that waist circumference (WC), a proxy for central adiposity, should be included with BMI in a ‘matrix’ to classify health risks.^7^ Waist circumference is regarded as a better measure of abdominal obesity,^8,9^ and the preference for using WC in most of the recent research studies is because of its simplicity, sensitivity and precision as an indicator of central or abdominal obesity.^10,11,12^ Thus, the method is accurate in measurement, easy to administer to a large number of individuals, low cost and having increased predictive power, which is equivalent to the combined predictive efforts of BMI and waist-to-hip ratio (WHR).^13^

Additionally, in children and adolescents, WC is strongly associated with high abdominal fat, elevated blood pressure and insulin resistance.^12^ In children and teenagers, WC is a more reliable indicator of elevated insulin levels and changes in plasma triglycerides than BMI. It also shows a higher positive relationship with systolic and diastolic blood pressure.^14^ According to Yamanaka et al.,^15^ WC can be used for screening and identifying children at risk for metabolic disease early in life. Numerous research has found a correlation between children’s metabolic risk and WC cut points. Other measurements related to abdominal obesity are WHR and the waist-to-height ratio (WHtR). However, because of poor association with metabolic and cardiovascular risk factors compared to other clinical adiposity measurements, the use of WHR as a predictor of cardiovascular risk factors has decreased in recent years.^16^ On the other hand, WHtR is known to be a reliable measure of central obesity and helps predict cardiovascular risk factors^17,18,19^ and is also known to be a better predictor of diabetes as compared to BMI.^7^

Fredriksen et al.^20^ carried out a research study in Norway, measuring WC of 2271 school children comprising 6–12 year old boys and girls. The results showed that BMI, WC and WHR increased by age for both sexes, while WHtR decreased with age in girls. Boys between 10 years and 12 years had a larger WC than girls. Girls had more weight than boys, and the researchers concluded that WC and WHR were reliable tools for identifying abdominal or central obesity in children.^20^ Ugochukwu et al.^21^ measured 593 secondary school children between 10 years and 17 years old in rural and urban Nnewi, Southeast Nigeria. There were 289 boys and 304 girls. The researchers used WC and WHR measurements, and the results revealed that girls had more weight and BMI than boys, regardless of residence. Boys were slightly taller, while girls had higher WC and WHR measures than boys in rural and urban residences. The WC and WHR cut-offs vary by nation because of ethnicity, environmental and genetic variables.^17^

Not much is known about how common abdominal obesity is among South African primary school learners.^6^ A few studies have evaluated the prevalence of abdominal obesity among South African children, particularly those living in rural areas.^8^ Therefore, it is purely hypothetical as to whether abdominal obesity is common among primary school learners in rural South Africa. Ukegbu et al.^22^ conducted a research study in Western Cape, North West, Limpopo rural, KwaZulu-Natal, Limpopo urban and Mpumalanga with 4954 school children aged 10–14 years. There were 2406 boys and 2548 girls. The study used WC and WHR measurements to compare across the study sites. Girls had higher WC and WHR values than boys of all ages. Rural Limpopo learners had the lowest WC values compared to urban learners in other provinces. Girls had a higher BMI than boys in all provinces, both rural and urban. Height, weight, BMI, WC and WHR increase with age in all provinces.^22^

Waist circumference, WHR and WHtR are essential anthropometric measures for children and adults as they directly measure central adiposity. Rising central adiposity is related to increased morbidity and mortality from diabetes and heart disease.^23^ The current study aims to evaluate BMI, WC, WHR and WHtR and their respective classification regarding the risk to comorbidities among rural primary school learners aged 9–14 years in the Eastern Cape province, South Africa.

## Methods

### Study design

The researchers opted for a cross-sectional study design. Trained facilitators and data collectors were used for the data-collection process.

### Measures

The major anthropometric measurements that were required included stature (cm), weight (kg), WC (cm) and hip (cm). The techniques of the International Society for the Advancement of Kinanthropometry’s (ISAK) were used to measure these anthropometric measurements. The measurements were then used to calculate BMI (kg/m^2^), WHR and WHtR. The weight and stature were used to determine the BMI. Waist hip ratio was calculated by dividing WC by hip while the WHtR was calculated by dividing WC by stature.

### Sampling

A random sample comprising 459 primary school learners (194 boys and 265 girls) aged 9–14 years from 12 schools in the Eastern Cape province of South Africa was used for the study. Initially, two out of six provincial districts in the Eastern Cape were purposely selected. From these districts, 12 rural primary schools were chosen randomly from a database from the Eastern Cape Department of Education. The schools were initially arranged alphabetically and then grouped according to district municipality. Only six randomly selected schools were taken from each group. Because of the age requirement of 9–14 years, only Grades 5–7 were selected to participate in the study. The researchers collected the class registers from the class teachers to select the eligible participants. The researchers considered even numbers from the class registers to select the participants. It is important to note that the class registers are arranged alphabetically using learners’ surnames. This is a standard form of how registers are ordered in public schools. A 10th of the selected number of learners was sampled to partake in the study based on the availability of research resources, and the researchers had much confidence in achieving the required minimum sample size from the employed approach. The selected learners were then issued consent forms to be signed by their parents or guardians.^24^

### Statistical analysis

The study used the Statistical Package for the Social Sciences (SPSS) version 29 for data analysis. A descriptive approach was adopted to describe and present the demographic and anthropometric characteristics of the respondents. To assess the gender (boys and girls) differences on anthropometric measurements, the independent samples *T*-Test was used. The one-way analysis of variance (ANOVA) test was adopted to assess the differences in anthropometric measurements by age (a categorical variable with three levels).

### Ethical considerations

Ethical clearance to conduct this study and permission to collect data was granted by the University of Fort Hare’s Ethics Committee (certificate reference number: LYO011SGOM01). Additionally, the researchers obtained approval from the Department of Education and the Department of Health of the Eastern Cape province, South Africa, to carry out the study. As the study was focused on minors, selected learners were given parental consent forms to seek permission from their parents or guardians to participate.

## Results

In Table 1, the results show that most of the 459 participants were girls (*n* = 265; 57.70%). The average age of the sample was 11.20 ± 1.60 years, with those aged between 11 years and 12 years constituting the greater proportion of the participants (*n* = 198; 43.10%). The average weight was 38.81 kg, with a standard deviation of 10.49. On average, the sampled participants were 144.16 (standard deviation [s.d.] = 10.37) cm tall with an average BMI of 18.41 kg/m^2^ (s.d. = 3.19). The results also showed an average WC of 62.10 cm (s.d. = 8.12) with an average WHR of 0.82 (s.d. = 0.15). On average, the samples’ WHtR was 0.44 (s.d. = 0.05).

**TABLE 1:** Biographic and anthropometric characteristics of respondents (*N* = 459).

Characteristic	*n*	%	Mean ± s.d.
**Gender**			
Boys	194	42.30	-
Girls	265	57.70	-
Age (in years)	-	-	11.20 ± 1.60
**Age categories**	-	-	-
9–10 years	115	25.10	-
11–12 years	198	43.10	-
13–14 years	146	31.80	-
Weight (in kg)	-	-	38.81 ± 10.49
Height/stature (in cm)	-	-	144.16 ± 10.37
BMI (in kg/m^2^)	-	-	18.41 ± 3.19
Waist circumference (in cm)	-	-	62.70 ± 8.12
Waist-hip ratio	-	-	0.82 ± 0.15
Waist-to-height ratio	-	-	0.44 ± 0.05

BMI, body mass index; s.d., standard deviation.

Table 2 presents the independent samples *T*-Test comparisons of the anthropometric measurements by gender. The test revealed no statistically significant differences in participants’ height by gender (*p* = 0.464). However, statistically significant differences were reported in weight (*p* = 0.014), BMI (*p* = < 0.001), WC (*p* = 0.039) and WHtR (*p* = 0.004). The mean levels showed that girls had statistically significantly higher measurements in all these anthropometric measurements than boys.

**TABLE 2:** Comparisons of anthropometric measurements by gender.

Anthropometric measurements	Boys (*n* = 194) (Mean ± s.d.)	Girls (*n* = 265) (Mean ± s.d.)	*P*
Height (cm)	144.57 ± 10.18	143.85 ± 10.51	0.464
Weight (kg)	37.47 ± 8.65	39.79 ± 11.57	0.014*
BMI (kg/m^2^)	17.72 ± 2.34	18.92 ± 3.62	< 0.001*
WC (cm)	61.82 ± 6.88	63.34 ± 8.88	0.039*
WHR	0.83 ± 0.12	0.82 ± 0.16	0.757
WHtR	0.43 ± 0.04	0.44 ± 0.05	0.004*

BMI, body mass index; WC, waist circumference; WHR, waist-hip ratio; WHtR, waist-to-height ratio; s.d., standard deviation.

* , *p*-value < 0.05, statistically significant differences.

In terms of the mean comparisons by age, the results in Table 3 revealed statistically significant differences for most of the anthropometric measurements (all *p* < 0.05) except for WHtR (*p* = 0.408). The post-hoc pairwise comparisons show that all the anthropometric measurements significantly increase by age of respondents except for WHR, where it generally decreases by age.

**TABLE 3:** Comparisons of anthropometric measurements by age of respondents.

Anthropometric measurements	9–10 years (*n* = 115) (Mean ± s.d.)	11–12 years (*n* = 198) (Mean ± s.d.)	13–14 years (*n* = 146) (Mean ± s.d.)	*P*
Height (cm)	135.39^c^ ± 6.91	142.69^b^ ± 7.18	153.85^a^ ± 7.66	< 0.001*
Weight (kg)	30.66^c^ ± 5.18	37.40^b^ ± 7.80	47.16^a^ ± 10.88	< 0.001*
BMI (kg/m^2^)	16.91^c^ ± 2.10	18.27^b^ ± 2.95	19.78^a^ ± 3.63	< 0.001*
WC (cm)	58.48^c^ ± 5.98	61.60^b^ ± 6.75	67.52^a^ ± 8.91	< 0.001*
WHR	0.85^a^ ± 0.21	0.81^b^ ± 0.07	0.82^a,b^ ± 0.16	0.028*
WHtR	0.44 ± 0.04	0.43 ± 0.05	0.44 ± 0.05	0.408

BMI, body mass index; WC, waist circumference; WHR, waist-hip ratio; WHtR, waist-to-height ratio; s.d., standard deviation.

a,b,c , Grouping for the one-way analysis of variance (ANOVA) post-hoc pairwise comparisons. Represents statistically significant different mean levels.

* , *p*-value < 0.05 statistically significant differences.

Table 4 presents reference categories of body weight ranges for BMI, WC, WHR and WHtR for adolescents, which is the age range of participants in this study. Using Table 4 as a guide, Table 5 shows the classification of anthropometric measurements in identifying those at risk of central adiposity-related comorbidities. Using BMI, 1.1% (*n* = 5) of the participants were obese and at high risk of comorbidities. Most participants were classified under low risk of comorbidities, while only 2.8% were overweight and at increased risk of comorbidities. Regarding classification using WC, only 0.4% (*n* = 2) of the primary school learners had high central fat and were at high risk of comorbidities. This was followed by only one participant (0.2%) classified under moderate central fat and at increased risk of comorbidities. Most participants (*n* = 456; 99.3%) were at low risk of comorbidities. The results on WHR show that 13.7% (*n* = 63) had high central fat and were at high risk of comorbidities. Almost a similar number of participants (*n* = 70; 15.3%) were classified as having moderate central fat, of which the majority were girls (*n* = 66). Using WHR, 71.0% of the participants were classified as having normal central fat and at low risk of comorbidities. Lastly, using WHtR, 10.7% of the primary school learners had high central fat and were at high risk of comorbidities. Contrary to other anthropometric measurement classifications, WHtR showed that most participants (*n* = 334; 72.4%) were classified as having moderate central fat and at increased risk of comorbidities. A significant number (*n* = 76; 16.6%) were reported as having normal central fat and at low risk of comorbidities.

**TABLE 4:** Classification of body weight according to anthropometric measurements.

Anthropometric measurements	Body weight		
	Normal weight	Overweight	Obese
**BMI (kg/m^2^)**			
Boys and girls (cut-off points)	18.5 – 24.9	25.0 to 29.9	≥ 30
**Waist circumference (cm)**			
Boys *(cut-off points)*	< 94	94 to 101.9	≥ 102
Girls *(cut-off points)*	< 80	80 to 87.9	≥ 88
**Waist-hip ratio**			
Boys *(cut-off points)*	≤ 0.95	0.96 to < 1.00	≥ 1.00
Girls *(cut-off points)*	≤ 0.80	0.81 to 0.85	≥ 0.86
**Waist-to-height ratio**			
Boys and girls *(cut-off points)*	< 0.4	0.4 to < 0.5	≥ 0.5

BMI, body mass index.

**TABLE 5:** Classification of anthropometric measurements and risk of central adiposity-related comorbidities.

Anthropometric measurements	Risk of comorbidities			
	Very low	Low	Increased	High
**BMI (kg/m^2^)**				
Boys and girls (*cut-off points*) (kg/m^2^)	< 18.5	18.5 to 24.9	25.0 to 29.9	≥ 30
*n*	297	144	13	5
%	64.7	31.4	2.8	1.1
Classification	Underweight	Normal weight	Overweight	Obese
**Waist circumference (cm)**				
Boys (*cut-off points*) (cm)	-	< 94	94 to 101.9	≥ 102
*n*	-	194	0	0
%	-	100.0	0.0	0.0
Girls (*cut-off points*) (cm)	-	< 80	80 to 87.9	≥ 88
*n*	-	262	1	2
%	-	98.9	0.4	0.8
Total (boys and girls)				
*n*	-	456	1	2
%	-	99.3	0.2	0.4
Classification	-	Normal central fat	Moderate central fat	High central fat
**Waist-hip ratio**				
Boys (*cut-off points*)	-	≤ 0.95	0.96 to < 1.00	≥ 1.00
*n*	-	184	4	6
%	-	94.8	2.1	3.1
Girls (*cut-off points*)	-	≤ 0.80	0.81 to 0.85	≥ 0.86
*n*	-	142	66	57
%	-	53.6	24.9	21.5
Total (boys and girls)				
*n*	-	326	70	63
%	-	71.0	15.3	13.7
Classification	-	Normal central fat	Moderate central fat	High central fat
**Waist-to-height ratio**				
Boys and girls (*cut-off points*)	-	< 0.4	0.4 to < 0.5	≥ 0.5
Boys	-			
*n*	-	32	147	15
%	-	16.5	75.8	7.7
Girls	-			
*n*	-	44	187	34
%	-	16.6	70.6	12.8
Total (boys and girls)				
*n*	-	76	334	49
%	-	16.6	72.4	10.7
Classification	-	Normal central fat	Moderate central fat	High central fat

BMI, body mass index.

## Discussion

This study evaluated BMI, WC, WHR and WHtR among rural primary school children aged 9–14 years in Eastern Cape province, South Africa. Our findings revealed that, on average, the BMI is generally considered underweight (18.41 kg/m^2^ ± 3.19 kg/m^2^). This result supports the findings reported by Violet et al.,^25^ who also reported a generally underweight sample (17.4 kg/m^2^ ± 3.35 kg/m^2^) in a cross-sectional study conducted in a South African rural setting with primary school children aged 9–12 years. However, our result is contrary to that of Matsha et al.,^26^ who had, on average, a BMI of 20.5 kg/m^2^ ± 4.4 kg/m^2^, which generally indicates normal body weight. Their study was conducted among 1272 randomly selected learners, aged 10–16 years, comprising 446 black African learners, 696 mixed-ancestry learners and 130 Caucasians learners in both urban and rural settings of South Africa. The differences in ethnicity and setting could explain the differences that our study has compared to Matsha et al.^26^ However, BMI is a commonly used measure of body fat and does not capture abdominal obesity. It has been suggested that WC and related measures for central adiposity, such as WHR and WHtR, should be included together with BMI.

Our study revealed that girls reported significantly higher BMI, WC and WHtR measurements. Regardless of setting, girls have been reported as having higher BMI, WC and WHR values than boys.^21,22^ However, other studies have reported significantly higher levels of BMI^25^ and WHR^26^ in boys than in girls. Contrary to this, our study reported no significant differences in WHR by gender. Our study also reported that all the anthropometric measurements significantly increase by the age of respondents except for WHR, which generally decreases with age. Ukegbu et al.^22^ also reported that height, weight, BMI, WC and WHR increase with age among school children aged 10–14 years across several provinces in South Africa. Regarding central fat accumulation, our study showed that when using WC, both boys (61.82 cm ± 6.88 cm) and girls (63.34 cm ± 8.88 cm) have normal central fat accumulation. The classification was somewhat different when using WHR, where, on average, boys (0.83 ± 0.12) were regarded as having normal central fat accumulation, whereas girls (0.82 ± 0.16) were, on average, regarded as having moderate central fat accumulation. Further, based on WHtR, our findings showed that, on average, both boys and girls had moderate central fat accumulation.

The notable outcome from this study was that, based on WHtR, the results revealed the acceptable ability of this measurement to classify children according to abdominal obesity or central adiposity, hence knowing their risk of comorbidities. The inferior performance of BMI in classifying children according to abdominal obesity is because BMI is not sensitive to body fat distribution, especially central adiposity.^27,28^ In our study, when using BMI and WC classifications, both genders are, on average, at low risk of comorbidities, but when using WHR, boys are at low risk, while girls are at increased risk of comorbidities. However, when using WHtR, both boys and girls are, on average, considered to be at increased risk of comorbidities. The excellent performance of WHtR can be attributed to the fact that it is known to be a reliable measure of central obesity and helps predict cardiovascular risk factors.^17,18^ Body mass index is not sensitive to central obesity,^27,28^ while the WHR’s clinical value is unreliable because of its poor relationship with metabolic and cardiovascular risk factors compared to other central adiposity measurements. Further, several studies have reported that WHtR is a better predictor of comorbidities risk than BMI and WHR.^7,19^

In this present study, the contributing factors to high levels of central obesity include, among others, physical inactivity, sedentary behaviour and household physical activity-related chores.^29^ Gomwe et al.^29^ reported that primary school learners had low levels of physical activity (PA) and high engagement in sedentary behaviour. This means that the primary school children did not meet the health-based guidelines for PA and sedentary behaviour, which adversely affects children’s health, growth and development. The low levels of PA can be attributed to the lifestyle in which most learners use scholar transport to go to school rather than walking or cycling. Further, most learners, particularly girls, prefer indoor activities or spend time on household chores, which causes them to have lower levels of PA than boys.^30^ In addition, the Department of Education in South Africa replaced physical education (PE) with life orientation (LO), and this is not assisting in motivating primary school learners to participate in physical activities.^29^ It is widely believed that declining levels of PA have affected the physical fitness levels of learners, and this decline has significantly contributed towards several health risks like hypertension, obesity, diabetes mellitus and chronic heart diseases.^31^

Data from several studies suggest that central obesity is a complex disorder impacted by a variety of factors, including hereditary, demographic and lifestyle factors.^32,33,34^ Hereditary and demographic characteristics such as sex, age and ethnicity cannot be modified. In contrast, lifestyle factors such as physical inactivity and unhealthy dietary habits are often preventable through some theory-based interventions.^32^ In the South African context, such interventions, especially those based on social cognitive theory and the theory of planned behaviour and integrated with formative research with targeted populations, have effectively increased self-reported adherence to physical activity guidelines among students in South Africa.^35^ Additionally, food outlets that sell ultra-processed foods can form part of the solution to address and help reduce the availability and promotion of unhealthy food options to primary school learners.^36^ These interventions aim to empower learners with the necessary knowledge and skills to make healthier food choices and engage in more active lifestyles, ultimately working to reverse the trend of central obesity.

### Limitations of the study

A significant limitation of this study is its inability to identify the factors leading to the prevalence of abdominal obesity among the sampled participants. Therefore, future research studies in South Africa should focus on understanding what factors influence the rising incidence and what interventions can best be made to reverse this trend of central obesity.

## Conclusion

To conclude, the notable results from this research study revealed that WC-based anthropometric measurements, especially WHtR, play a significant role in identifying those at risk of central adiposity-related comorbidities. This ability of WHtR to capture central adiposity and its association with the risk of comorbidities among children shows the reliability of this measure in assessing abdominal obesity among children. To prevent the risks of metabolic diseases in childhood, it is necessary to detect central obesity early using WC-related anthropometric measurements as key indicators, especially WHtR. To reverse the trend of central obesity, it is necessary to develop interventions aimed at increasing physical activity, promoting healthier lifestyles and promoting education on dietary habits among primary school learners in South Africa.
